# Suture granuloma with hydronephrosis caused by ileostomy closure after rectal cancer surgery: a case report

**DOI:** 10.1186/s40792-021-01303-7

**Published:** 2021-09-18

**Authors:** Yasuhiro Takano, Koichiro Haruki, Shu Tsukihara, Tadashi Abe, Muneyuki Koyama, Daisuke Ito, Hironori Kanno, Kyonsu Son, Nobuyoshi Hanyu, Ken Eto

**Affiliations:** 1 Department of Surgery, Tokyo General Hospital, 3-15-2, Ekoda, Nakano-ku, Tokyo, 165-8906 Japan; 2 Department of Surgery, The Jikei University School of Medicine, 3-25-8, Nishi-Shimbashi, Minato-ku, Tokyo, 105-8461 Japan

**Keywords:** Suture granuloma, Hydronephrosis, Ileostomy closure, Rectal cancer

## Abstract

**Background:**

Suture granuloma with hydronephrosis after abdominal surgery is extremely rare. We herein report a successfully treated case of suture granuloma with hydronephrosis caused by ileostomy closure after rectal cancer surgery.

**Case presentation:**

A 63-year-old male underwent laparoscopic low anterior resection with covering ileostomy. Two months after primary operation, ileostomy closure was performed with two layered hand-sewn suture (Albert–Lembert method) using absorbable suture. In that operation, marginal blood vessels in the mesentery were ligated with silk suture. The patient had remained in remission with no evidence of tumor recurrence, however, 2 years and 5 months after primary surgery, a contrast-enhanced computed tomography (CT) scan showed a mass-forming lesion on the right external iliac artery (43 × 26 mm) and hydronephrosis. Positron emission tomography/computed tomography (PET/CT) showed a mass-forming lesion without high accumulation, which obstructed the right ureter. Recurrence could not be ruled out due to the rapid appearance of tumor and hydronephrosis in the short-term period. Thus, the patient underwent laparotomy. The tumor located in the mesentery near the anastomosis of ileostomy closure and it was strongly adherent to the retroperitoneum, which obstructed the right ureter. The adhesion between the tumor and ureter was carefully dissected and tumor resection with partial small bowel resection was then performed with preservation of the ureter using ureteral stents. Pathological examination of the tumor revealed fibrous proliferation of foreign body granuloma. In the resected tumor, sutures with foreign giant cells were found. Therefore, we diagnosed the tumor as silk suture granuloma, which was caused by the silk suture used to ligate blood vessels of the mesentery at the ileostomy closure. The patient remained well with no evidence of tumor recurrence as 5 years after the primary operation of rectal cancer.

**Conclusions:**

Suture granuloma is a rare surgery-related complication in the postoperative surveillance of patients with colorectal cancer. If suture granuloma mimicking local recurrence is a differential diagnosis, it would be important to consider to avoid unnecessary extended resection.

## Background

Foreign body granuloma is a rare benign tumor, which is induced by medical materials several months or years after surgery [1]. Particularly, suture is well recognized as one of the major causes of granuloma [2]. In cases of post-surgery of malignant tumors, to differentiate suture granuloma from recurrence or metastasis is critically important for appropriate treatment.

Hydronephrosis is one of the suggestive of recurrence, which can be caused by lymph node metastasis or peritoneal recurrence in patients who underwent colorectal cancer surgery [3]. It is very rare that suture granuloma developed hydronephrosis after rectal cancer surgery.

We herein report a successfully treated case of suture granuloma with hydronephrosis by tumor resection.

## Case presentation

**Fig. 1:**
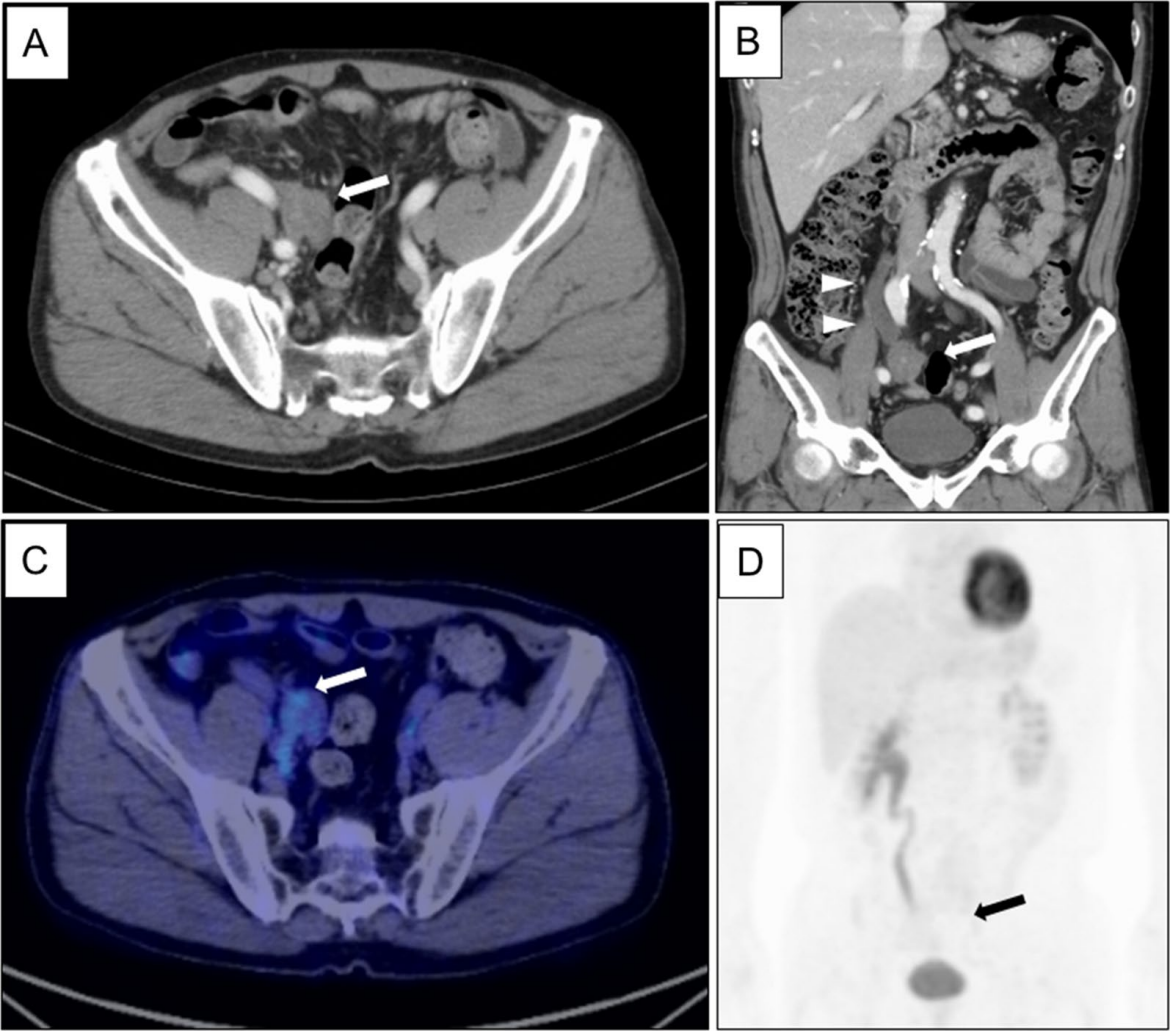
Contrast-enhanced computed tomography (CT) and positron emission tomography/computed tomography (PET/CT). Contrast-enhanced computed tomography (CT) scan showed a mass-forming lesion on the right external iliac artery (43 × 26 mm) (**A** arrow, **B** arrow) and hydronephrosis (**B** arrowhead). Positron emission tomography/computed tomography (PET/CT) showed a mass-forming lesion without high accumulation (**C** arrow), which obstructed the ureter (**D** arrow)

**Fig. 2:**
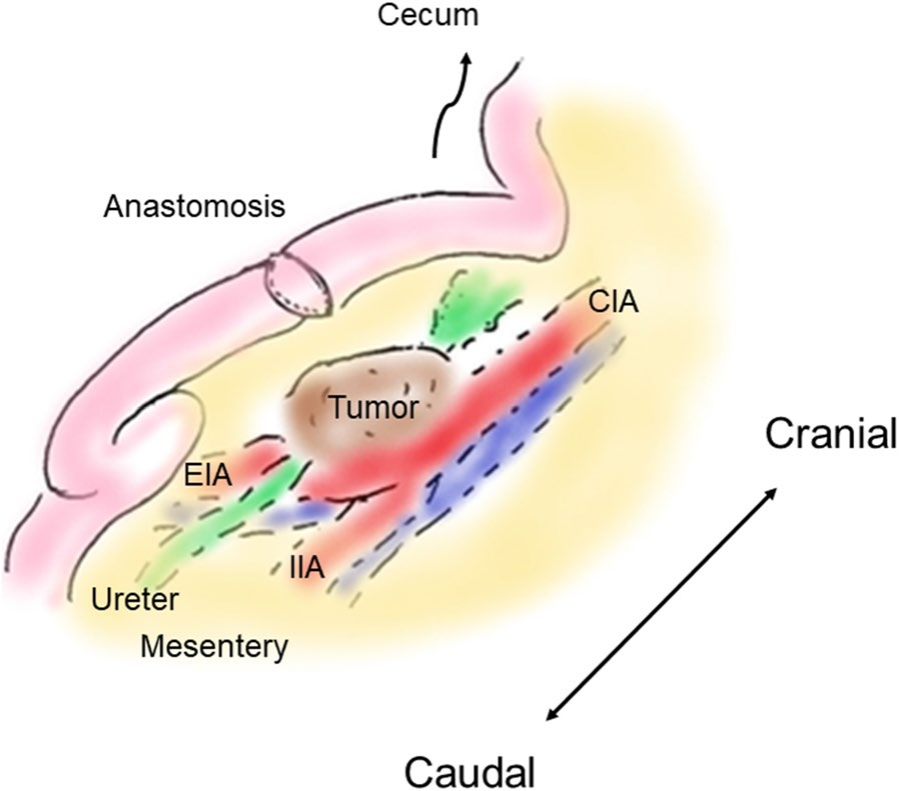
Intraoperative figure. The tumor was located in the mesentery near the anastomosis of ileostomy closure and it was strongly adherent to the retroperitoneum, which obstructed the right ureter. *CIA* common iliac artery, *EIA* external iliac artery, *IIA* internal iliac artery

**Fig. 3:**
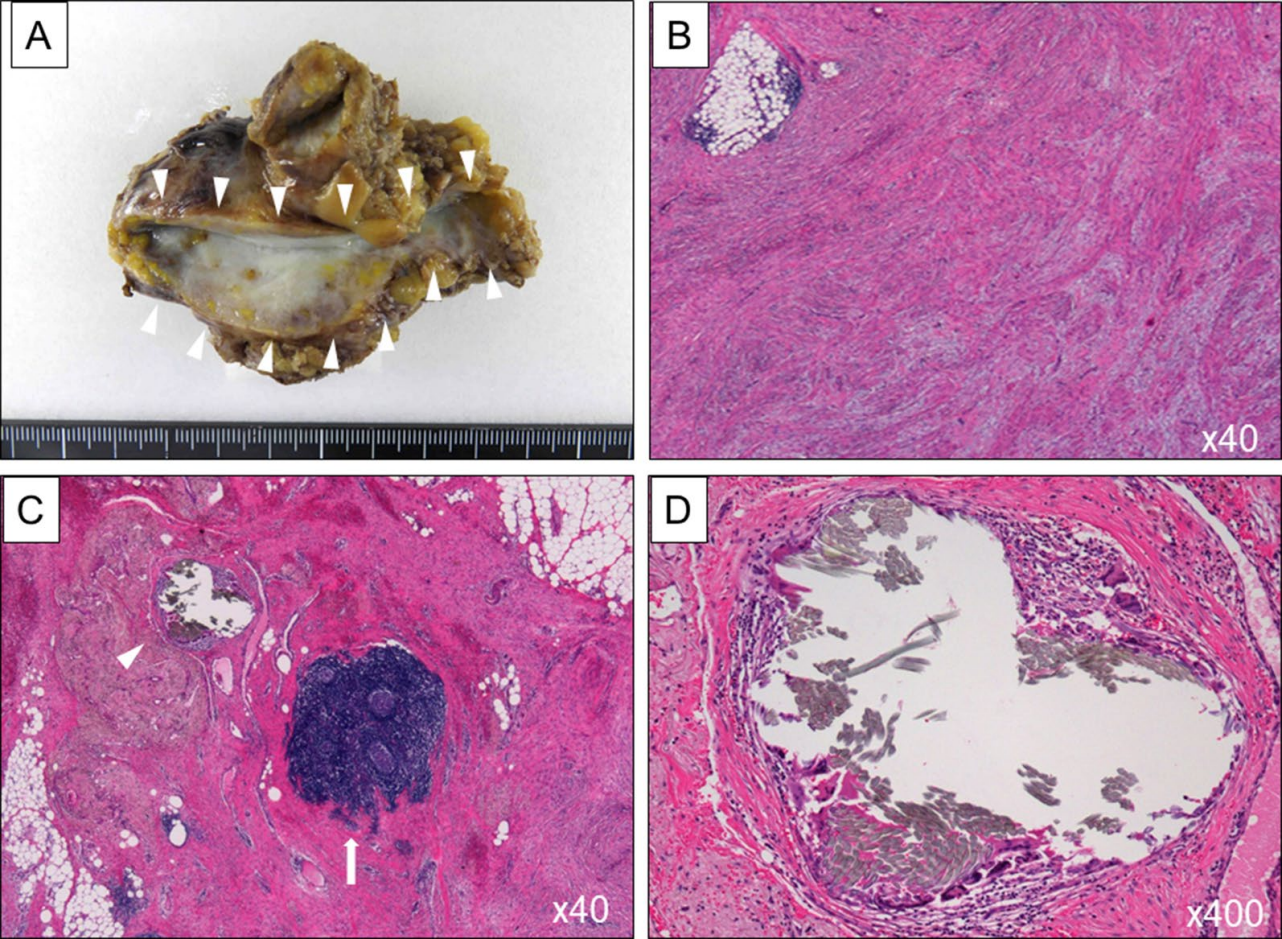
Resected specimen and pathological examination. The resected specimens revealed a mass that had surrounded the right ureter (**A** arrow). Pathological examination of the tumor revealed dense fibrous proliferation (**B**) and lymphatic follicles with germinal centers (**C** arrow). In the partially resected tumor, sutures with foreign giant cells were found (**C** arrowhead, **D**)

A 63-year-old male was admitted to undergo colonoscopy for the evaluation of blood feces. The patient underwent endoscopic resection for a type Isp polyp of the lower rectum. The pathological diagnosis examination revealed well-differentiated adenocarcinoma with submucosal layer (2500 µm) invasion and lymphatic infiltration (ly1). The patients underwent laparoscopic low anterior resection with covering ileostomy as additional resection. The pathological examination of surgical specimen revealed there was no lymph node metastasis, resulting in pStage I. Two months after primary operation, ileostomy closure was performed with two layered hand-sewn suture (Albert–Lembert method) using absorbable suture. In that operation, marginal blood vessels in the mesentery were ligated with silk suture. After ileostomy closure, the patient had been followed by a contrast-enhanced computed tomography (CT) every 6 months and tumor marker every 3 months, and remained in remission with no evidence of tumor recurrence. However, 2 years and 5 months after the operation, CT showed a mass-forming lesion on the right external iliac artery (43 × 26 mm) (Fig. 1A) and hydronephrosis (Fig. 1B). Positron emission tomography/computed tomography (PET/CT) showed a mass-forming lesion without high fluorodeoxyglucose (FDG) accumulation (Fig. 1C), which obstructed the right ureter (Fig. 1D). Tumor markers were as follows: carcinoembryonic antigen (CEA) 3.6 ng/ml; carbohydrate antigen 19-9 (CA19-9) 3.0 U/ml and there were no findings suggestive of metastasis nor recurrence in other organs. Although PET/CT findings and tumor marker were not positive, recurrence could not be ruled out due to the rapid appearance of tumor and hydronephrosis in the short-term period. Thus, the patient underwent laparotomy. The tumor was observed in the mesentery near the anastomosis of ileostomy closure and it was strongly adherent to the retroperitoneum, which obstructed the right ureter (Fig. 2). The adhesion between the tumor and ureter was carefully dissected and tumor resection with preservation of the ureter using ureteral stents. After tumor resection, partial small bowel resection was performed due to disturbance of mesenteric blood flow. The resected specimens revealed a mass-forming lesion surrounded the right ureter (Fig. 3A). Pathological examination of the tumor revealed dense fibrous proliferation and lymphatic follicles with germinal centers (Fig. 3B, C). In the resected tumor, sutures with foreign giant cells were found (Fig. 3C, D). Therefore, we diagnosed the tumor as suture granuloma, which was caused by the silk suture using to ligate blood vessels in mesentery at the ileostomy closure. After operation, the patient was discharged on postoperative day 14 without any complications. The patient remained well with no evidence of tumor recurrence as 5 years after the primary operation of rectal cancer.

## Discussion

Suture granulomas are caused by persistent foreign body reactions to suture material due to their antigenicity and/or presence of bacterial infection in the fibers of silk [4, 5]. Silk sutures are non-absorbable yarn which show a particularly high incidence of granuloma formation of 0.6–7.1% [6, 7]. Because suture granuloma often grows rapidly in the short-term period [8, 9], differentiating from recurrent tumors after surgery of malignant tumors is difficult in postoperative malignancy surveillance [8, 9]. Suture granuloma has shown false positive on PET/CT in 70–80% of granuloma cases due to the rapid growth of the granulomas caused by inflammatory response [8]. In the current case, PET/CT did not show high FDG accumulation. The reason of negative findings might be that PET/CT was performed when the inflammatory activity was low. In cases of granuloma, the result on PET/CT may depend on the timing of tumor growth unlike malignant tumors. In addition, the granuloma that causes external compression of adjacent organs is extremely rare. Although there have been a few reports of anastomotic suture granulomas that obstructed the lumen of ureter or bronchus [10, 11], extramural compression by suture granuloma has not been well-described. Unlike malignant tumors, granuloma is often non-invasive [12, 14], therefore, in the current case, although the suture granuloma involved the right ureter, careful resection enabled to preserve ureter. Thus, if granuloma is a differential diagnosis, it would be important to consider to avoid unnecessary extended resection.

As to the granuloma after colorectal cancer surgery, seven case reports have been published in the English literature; details of the current case and the other seven cases are summarized in Table 1 [8, 12–16]. Only one patient (Case 2) among them had hydronephrosis by granuloma in the right pelvic side peritoneum 2 years and 6 months later after rectal cancer surgery [12]. There have been no reports of granuloma with hydronephrosis in the other pelvic surgery. In the reports on colorectal cancer, the most frequent causal material was suture (five patients), followed by metal staple, clip and food residues. In the current case, the suture granuloma was developed from the silk suture that was used at the time of ileostomy closure.

**Table 1:** Cases of granuloma in patients who underwent colorectal cancer surgery

	Age	Sex	Primary diagnosis	Interval	Granuloma site	PET/CT	Size (mm)	Causal material
1 [8]	71	Male	Sigmoid colon cancer, liver metastasis	4 months	Liver	Positive	15	Suture (silk)
2 [12]	67	Male	Rectum cancer	30 months	Pelvic peritoneum	Positive	34	Suture (NA)
3 [12]	37	Male	Transverse colon cancer, liver metastasis	3 months	Liver	Positive	NA	NA
4 [13]	85	Male	Sigmoid colon cancer	10 months	Abdominal wall (port site)	Positive	20	Suture (absorbable suspected)
5 [14]	66	Male	Sigmoid colon cancer, liver metastasis	29/19 months	Liver	NA	23/45	Metal staples/clip
6 [15]	31	Female	Left side colon cancer (perforation)	7 months	Liver	Positive	NA	Food residues
7 [16]	60	Male	Ascending colon cancer	24 months	Mesentery	NA	40	Suture (silk)
Current case	63	Male	Rectum cancer	29 months	Mesentery	Negative	43	Suture (silk)

*NA* not available

In colorectal cancer surgery, silk suture has been reported to develop surgical site infection more frequently compared to absorbable suture [17]. In addition, the occurrence rates of granuloma by silk suture have been higher than those by absorbable suture [7]. In the present era of modern surgical practice, the use of silk sutures is decreasing and is even rarer in laparoscopic surgery. However, it should be noted that silk sutures used to ligate blood vessels may be risks of granuloma occurring in traditional open surgery or extra-abdominal procedures of laparoscopic surgery. Therefore, silk sutures should be avoided if possible, considering the higher incidence of surgical site infection as well as suture granuloma.

## Conclusions

We reported to our knowledge the case of suture granuloma with hydronephrosis caused by ileostomy closure after rectal cancer surgery.

## Data Availability

The dataset supporting the conclusion of this article is included within the article.

## Abbreviations

CIA: Common iliac artery
CT: Computed tomography
EIA: External iliac artery
FDG: Fluorodeoxyglucose
IIA: Internal iliac artery
PET/CT: Positron emission tomography/computed tomography
